# Comparative Study of Two Pose Measuring Systems Used to Reduce Robot Localization Error

**DOI:** 10.3390/s20051305

**Published:** 2020-02-28

**Authors:** Marek Franaszek, Geraldine S. Cheok, Jeremy A. Marvel

**Affiliations:** National Institute of Standards and Technology, Gaithersburg, MD 20899, USA; cheok@nist.gov (G.S.C.); jeremy.marvel@nist.gov (J.A.M.)

**Keywords:** pose measuring systems, robot localization error, volumetric error compensation, accuracy, rigid-body registration.

## Abstract

The performance of marker-based, six degrees of freedom (6DOF) pose measuring systems is investigated. For instruments in this class, the pose is derived from locations of a few three-dimensional (3D) points. For such configurations to be used, the rigid-body condition—which requires that the distance between any two points must be fixed, regardless of orientation and position of the configuration—must be satisfied. This report introduces metrics that gauge the deviation from the rigid-body condition. The use of these metrics is demonstrated on the problem of reducing robot localization error in assembly applications. Experiments with two different systems used to reduce the localization error of the same industrial robot yielded two conflicting outcomes. The data acquired with one system led to substantial reduction in both position and orientation error of the robot, while the data acquired with a second system led to comparable reduction in the position error only. The difference is attributed to differences between metrics used to characterize the two systems.

## 1. Introduction

The pose of a rigid object is defined by its location and orientation in three-dimensional (3D) space. A location is determined by a 3×1 vector and the orientation by a 3×3 rotation matrix, which can be parametrized by three angles (for example, Euler angles or yaw, pitch and roll). In most practical applications, this six degree of freedom (6DOF) data is not directly measured, but rather is derived from other raw measurements followed by some error minimization procedures. The procedure for accomplishing this depends on the sensor used and the choice of post-processing algorithm(s). For example, many sensor systems can acquire 3D point clouds quickly, and fit the data to one or more objects’ computer-aided design (CAD) models using different versions of the Iterative Closest Point (ICP) algorithm [1]. For instance, data acquired with a red-green-blue-depth (RGB-D) camera can be processed using a voting scheme that matches pose estimates for an unstructured bin picking application [2]. An approach based on pose-from-silhouette can be used to get a 6DOF pose from two-dimensional (2D) images [3,4]. In image-guided surgery, 6DOF pose of a surgical tool can be tracked by a camera based system using spherical markers that are attached to the tool [5].

In practical applications, it is important to know the accuracy and repeatability of the robot’s pose. Many measurement systems can rapidly acquire repeated measurements, filter out noise, and return mean values of individual 3D points. These preprocessing steps substantially reduce the uncertainty of the derived pose. However, a reliable estimate of the systematic pose error is a more difficult task. It requires comparisons of 6DOF poses as measured by an instrument under test (IUT) to ground truth (GT) measurements. Since the GT sensor and the IUT acquire data in two different coordinate frames, a transformation between both frames must be calculated. Thus, the error defined as the difference between the IUT and the GT pose includes a registration error. To minimize the registration error, the relative pose in the GT frame is compared to a corresponding relative pose in the IUT frame. This may be achieved by means of the ASTM E2919-13 standard for evaluating the performance of static 6DOF pose measuring systems [6].

Since the 6DOF pose is derived from processing multiple 3D points, it is important to understand how noise and bias in the points propagate to the derived pose. For many pose measuring systems, no explicit formula is available. However, for a class of systems that derive pose by measuring several markers, extensive research has yielded analytical formulas that link the uncertainty and bias in the marker measurements with the uncertainty and error of the derived 6DOF pose. Such marker-based systems estimate pose by performing point-based, rigid-body registrations between two sets of corresponding points. Closed-form equations for propagating the uncertainty of the measured markers to the pose were derived in [7,8] while the influence of bias in the marker locations on the pose was studied in [9]. The analytical formulas derived in these papers for different noise models were tested in both computer simulations and camera-based tracking systems [10,11]. The formulas enabled the optimization of marker placement so that the error propagated to a specific point using noisy, inaccurate registration could be minimized [12]. This is especially important in medical applications [13,14,15], and was used to design optically-tracked instruments for image-guided surgery [5].

In this paper, new metrics gauging the performance of marker-based, pose measuring systems are proposed that do not require a GT measurement system. The metrics gauge how well the rigid-body assumption (central to the registration procedure) is preserved, with the premise being that, if the rigid-body condition is better preserved, the system will yield a better pose measurement. The value of these metrics is demonstrated on the problem of reducing robot localization error using an external pose measuring system. The Restoration of Rigid Body Condition (RRBC) procedure [16] was used to reduce both the position and the orientation errors of the robot end effector. The procedure was tested on simulated 6DOF data as well as on a physical robot arm, using 6DOF data acquired by two different pose measurement systems. The results showed that the procedure worked significantly better for one measurement system than it did for the other. The metrics introduced in this paper have the added benefit of explaining this difference in performance, as corresponding metrics for both systems differ by an order of magnitude. The proposed metrics are easy to calculate from the data acquired by marker-based, pose measuring systems, and may be useful in determining if a given system is suitable for a particular task.

The paper is organized as follows: A short review of methods to reduce robot localization error is provided in Section 2. This is followed in Section 3 by a brief description of the RRBC procedure, and the metrics gauging performance of marker-based, pose measuring systems are defined in Section 4. The experimental setup is then presented in Section 5, followed by results and discussion in Section 6 and Section 7, respectively.

## 2. Reduction of Robot Localization Error

Most robotic tasks in manufacturing require precise manipulation and placement of an end-of-arm tooling. For example, automated drilling in the aerospace applications must satisfy a position error of the drill bit less than 0.25 mm [17]. Errors in positioning the end-of-arm tooling are typically associated with localization accuracy. Localization errors for industrial robots have two principal causes: (1) incorrect values of the Denavit-Hartenberg (DH) parameters in the robot’s kinematic model; and (2) other, non-kinematic sources of error such as thermal effects, backlash, friction, drift, joint compliance, or deformation under gravity. For robots with revolute joints, incorrect values of joint-offsets contribute roughly 80% of the total robot localization error [18,19]. This kind of error is most frequently minimized by remastering the robot. For many, low-precision applications, remastering the robot may be achieved by visually aligning dial gauges mounted at each joint and running a vendor-provided program. Higher-precision remastering techniques often require external sensors for more accurate measurements. Such setups often consist of a spherically mounted retroreflector (SMR) mounted on the robot’s tool flange. The SMR is then moved by the robot to many locations within the robot’s working volume. A high-precision measurement system (e.g., a laser tracker) collects 3D points at each pose, and the kinematic model parameters are obtained by minimizing the distances between measured points and corresponding points derived from the model [20,21].

To further reduce any residual errors remaining after calibration—as well as other, non-kinematic errors—different compensation techniques have been developed. Many of these techniques require dynamic tracking of the robot’s movement by external, vision-based systems, as in video servoing [22,23] or different versions of the Volumetric Error Compensation (VEC) procedures [24,25,26,27,28,29]. Such approaches have been demonstrated to reduce the position error to 0.05 mm, and orientation error to 0.05° for industrial robots integrated with an optical coordinate measuring machine [30]. Another approach used an indoor global positioning system (iGPS) to reduce position and orientation errors tenfold to 0.15 mm and 0.02°, respectively [31].

Even after remastering, a robot may still exhibit significant pose error as a result of non-kinematic causes. As such, another approach to recalibration generates static, pose-correction maps to correct for kinematic errors without having to measure DH parameter offsets. Corrections to the robot’s pose are calculated in predetermined locations from data acquired before regular robot operations begin. These corrections are then interpolated to get the correction for an arbitrary robot pose during regular operations. Different interpolation procedures have been used. For example, trilinear, cubic, and fuzzy interpolation were tested in computer simulations [32]. The kriging interpolation, frequently used in geostatistics [33], was applied to reduce position error of a drilling end effector using a SMR and a laser tracker, resulting in an average position error reduction to 0.106 mm [34]. A similar procedure was applied to reduce the position error of a drilling and riveting end effector mounted on a robot arm; the reported maximum absolute position error was reduced to 0.32 mm [35]. In both studies, the corrections applied to the commanded robot locations were calculated from experimentally-determined semivariograms [36]. No corrections to the end effector orientations were calculated in either study.

Another method that does not require dynamic tracking, and that can reduce both position and orientation error, was introduced in [16]. The Restoration of Rigid Body Condition (RRBC) was tested on both simulated 6DOF data and data acquired by a motion capture system tracking an industrial robot arm. The outcome of this study was ambiguous. The method worked very well on simulated data and resulted in a 97% reduction in the median position error (down to 0.29 mm) and 99% reduction in the median orientation error (down to 0.01°). However, for experiments using the robot arm, only the reduction in position error matched the performance observed in simulation (97%, median reduced error of 0.3 mm). The reduction in the median orientation error was much smaller: only 57% (down to 0.27°) compared to 99% in simulation results.

In this paper, the RRBC method was used in another experiment using the same robot arm as in [16], but a different pose measuring system was used: a laser tracker. In this experiment, the method worked well for both position and orientation error (92% reduction in position error, down to 0.43 mm and 88% reduction in orientation error, down to 0.038°). The difference in the current and previous results is attributed to the substantially different characteristics of the pose measuring systems used in both experiments. Both systems derived 6DOF pose from the measurement of three 3D points which should preserve the rigid-body condition (i.e., the relative distances between points should be constant) to ensure correct, unbiased pose determination. Neither of the systems satisfies this requirement perfectly, but the deviations from the rigid-body condition are an order of magnitude smaller for the laser tracker measurements than for the motion capture system. Thus, contrary to our earlier conclusion in [16], the reduction in both position and orientation robot error is possible, provided that the bias in the 6DOF data used to calculate the corrections is sufficiently small.

## 3. Description of the RRBC Method

In the RRBC method, small corrections to the commanded robot poses (target poses) are linearly interpolated from previously determined corrections calculated from fiducial poses measured by a pose measuring system and derived from the robot kinematic model. Fiducial poses are measured in both robot and sensor frames while the target poses are usually only measured in the sensor frame but need to be accessed in the robot frame. To calculate the corrections, a rigid body transformation from the sensor frame to the tool center point (TCP) frame is needed.

Generally, when there is a constant offset transformation, $\hat{X}$, between the TCP frame and the robot’s tool flange, hand-eye calibration is needed to find the transformation, $\hat{Y}$, from the sensor’s coordinate system to the robot’s coordinate system. This procedure requires J≥3 different measurements of corresponding poses, ${\hat{B}}_{j}$, in the sensor’s coordinate system, and ${\hat{A}}_{j}$ in the robot’s coordinate system. Both homogeneous transformations, $\hat{X}$ and $\hat{Y}$, can be calculated by solving the set of equations:

(1) $${\hat{A}}_{j}\;X=\;Y\;{\hat{B}}_{j}$$

for all j=1,…, J. Equation (1) can be rewritten for the orientation and the position parts separately as

(2) $$A_{j}\;X=Y\;B_{j}\;,$$

(3) $$A_{j}\;x+\;a_{j}=Y\;b_{j}+y\;,$$

where $X,\;Y,\;A_{j},B_{j}$ are 3×3 rotation matrices, and $x,y,a_{j},b_{j}$ are column vectors. There are many different methods to solve Equations (2) and (3) for $\hat{X}$ and $\hat{Y}$. In this study, we use a modified analytical solution based on the Kronecker product developed in [37]. The original method does not guarantee that matrices X and Y are orthogonal. Therefore, we apply an orthogonalization procedure to the resulting matrices as described in [38]. After the homogeneous matrices, $\hat{X}$ and $\hat{Y}$, are determined, poses from the sensor frame {B} can be transformed to the robot frame {A}. Fiducial poses mapped from the sensor frame do not exactly match the corresponding measured fiducial poses in the robot frame. A small rotation matrix $\Lambda_{j}$ and a position vector $\lambda_{j}$ are calculated from Equations (2) and (3) as:

(4) $$\Lambda_{j}=Y\;B_{j}\;X^{T}\;A_{j}^{T},$$

(5) $$\lambda_{j}=Y\;b_{j}+y-\;\left(A_{j}\;x+\;a_{j}\right),$$

where ${\left(\ldots \right)}^{T}$ indicates a transposed matrix. Once all matrices $\Lambda_{j}$ and vectors $\lambda_{j}$ are calculated from the fiducial poses ${\hat{A}}_{j}$ and ${\hat{B}}_{j}$, they can be used to estimate the orientation and position corrections of a target pose, $\left(\tilde{A},\tilde{\alpha }\right)$, transformed to the robot frame from the sensor frame

(6) $$\Lambda_{\alpha }\left(\tilde{A},\tilde{\alpha }\right)=ort\left(\sum_{j=1}^{J}w_{j}\Lambda_{j}\right),$$

(7) $$\lambda_{\alpha }\left(\tilde{A},\tilde{\alpha }\right)=\;\sum_{j=1}^{J}w_{j}\lambda_{j\;},$$

where ort(…) denotes the orthogonalization procedure developed in [38], and $\left(\tilde{A},\tilde{\alpha }\right)$ is the target pose (B,β) transformed from the sensor frame to the robot frame using

(8) $$\tilde{A}=Y\;B\;X^{T},$$

(9) $$\tilde{\alpha }=Y\;\beta +y-\tilde{A}\;x\;.$$

The corrected target rotation, $\Lambda_{\alpha }\tilde{A}$, and the corrected target position, $\tilde{\alpha }+\;\lambda_{\alpha }$, should be closer to the actual (and usually unknown) rotation A and position α in the robot frame. When the target pose (A,α) is also measured in the robot frame (as in this research study), the corresponding target error can be calculated and used to gauge the performance of the error compensation procedure.

For the orientation error, we rely on the angle-axis (ρ,u) representation of the rotation, R(ρ,u). For the position error, we use the Euclidean norm δ of the vector. We ensure that ρ is always non-negative by using the property of rotation that R(−ρ,u)=R(ρ,−u). Thus, the target error has two components, (ρ,δ), which are evaluated for the uncorrected poses in the robot’s coordinate system using:

(10) $$R\left(\rho_{unc},u_{unc}\right)=\tilde{A}\;A^{T},$$

(11) $$\delta_{unc}=\;\|\tilde{\alpha }-\;\alpha \|,$$

as well as for the corrected poses using:

(12) $$R\left(\rho_{cor},u_{cor}\right)=\;\Lambda_{\alpha }\;\tilde{A}\;A^{T},$$

(13) $$\delta_{cor}=\;\|\tilde{\alpha }+\;\lambda_{\alpha }-\;\alpha \|.$$

The application of the RRBC method is successful when the corrected errors, $\left(\rho_{cor},\delta_{cor}\right)$, are smaller than their corresponding uncorrected errors, $\left(\rho_{unc},\delta_{unc}\right)$. Note that both the angular error, ρ, and the position error, δ, are invariant to the coordinate frame. Therefore, they are convenient metrics to gauge the performance of the RRBC method. Alternatively, the difference between the two 6DOF poses could be used as a performance gauge. However, this requires the comparison of twelve numbers that depend on the choice of coordinate system, whereas the invariants (ρ,δ) yield the same conclusion in a more concise way.

The weights, $w_{j}$, are used to calculate the orientation corrections in Equation (6), and position correction in Equation (7). We calculate the Euclidean distances, $d_{j}$, between the joint angles of the robot arm, $\theta_{t}$, corresponding to the target pose, and the joint angles of the *j*-th fiducial $\theta_{f,j}$:

(14) $$d_{j}=\;\sqrt{\sum_{n=1}^{N}{\left[\theta_{t}\left(n\right)-\theta_{f,j}\left(n\right)\right]}^{2}},$$

where θ(n) is the *n*-th joint angle (for target or fiducial configuration), and N is the total number of robot joints. Then, the inverse distance $d_{j}^{'}=1/d_{j}$ was used to calculate the weights as

(15) $$w_{j}=d_{j}^{'}/\sum_{j=1}^{J}d_{j}^{'}.$$

## 4. Performance Metrics

Two metrics are defined in this paper to gauge how well the rigid-body condition is preserved by marker-based pose measuring systems. Additionally, a third metric is defined which can be used to compare any two corresponding 6DOF datasets acquired in two different coordinate systems (for example, one dataset could be acquired by the GT and another by the IUT). In the context of reducing errors using the RRBC, we associate the first frame, {A}, with the robot, and the second frame, {B}, with the sensor. This third metric is sensitive to differences between two relative rotations in both coordinate systems. A fourth, auxiliary, metric is defined specifically for robotic applications, where both the robot and sensor errors contribute to a non-zero value of the third metric. Details of each metric are provided below.

### 4.1. Metric 1: Distances between Markers

The rigid-body condition requires that a distance, $L_{n,m}\left(j\right)$, between any two markers, n and m, is fixed and does not depend on the *j-*th pose of the rigidly-mounted markers. At least three markers must be used to uniquely define a 6DOF pose. To identify each marker given an arbitrary marker configuration, the layout of the markers should be such that the distances between the centroid of all markers and each individual marker are distinct, and can be easily sorted from the longest to the shortest (i.e., $L_{min}\left(j\right)<\ldots <L_{n}\left(j\right)<\ldots <L_{max}\left(j\right)$ for each j ). After collecting marker locations for many poses, j=1,…J, the standard deviation, $\sigma_{L}$, and the mean distance, $\bar{L}$, are calculated for each pair of markers, (n,m). Thus, the ratio $\sigma_{L}/\bar{L}$ can be used as the first metric to gauge the performance of any marker-based pose measuring system. For an ideal system that perfectly satisfies the rigid-body assumption, this metric is zero.

### 4.2. Metric 2: Angles of Deviation from the Mean Rotation

For marker-based systems, the pose is derived from N≥3 locations of 3D points. For N=3 (as is the case in this report), the three markers measured for the *j-*th pose are labeled as $b_{S,j},\;b_{M,j},\;$ and $b_{L,j}$, according to the three lengths $L_{S}<L_{M}<L_{L}$ described previously. Then, three unit column vectors, $\left(n_{1},n_{2},n_{3}\right)$, are used to define the orientation matrix, $B_{j}$, as:

(16) $$B_{j}=\;\left[\begin{array}{ccc} n_{1,x} & n_{2,x} & n_{3,x}\\ n_{1,y} & n_{2,y} & n_{3,y}\\ n_{1,z} & n_{2,z} & n_{3,y} \end{array}\right],$$

where:

(17) $$n_{3}=\;s_{3}\left(b_{L,j}-b_{M,j}\right)\times \left(b_{S,j}-b_{M,j}\right)\;,$$

(18) $$n_{2}=s_{2}\left(b_{L,j}-\;b_{j}\right),$$

(19) $$n_{1}=\;n_{3}\times n_{2},$$

and $s_{2},\;s_{3}$ are normalization factors ensuring that $n_{2},\;n_{3}$ are unit vectors, × indicates the vector product, and $b_{j}$ is the centroid of $b_{L,j},\;b_{M,j}$ and $b_{S,j}$.

For j=2,…,J, different pose measurements (all triplets of points $\left[b_{S,j},b_{M,j},b_{L,j}\right]$) can be registered to the first triplet, $\left[b_{S,1},b_{M,1},b_{L,1}\right]$, using a point-based rigid body transformation [39]. From the transformed triplets, the corresponding rotations, ${\tilde{B}}_{j}$, can be calculated using Equation (16), and the average rotation, ${\tilde{B}}_{avg}$, can be calculated as in [38]. Finally, small angles, $\psi_{j}$, of relative rotations $∆\tilde{B}\left(\psi_{j},u_{j}\right)$ between the average ${\tilde{B}}_{avg}$ and ${\tilde{B}}_{j}$ rotation are calculated. Angles $\psi_{j}$ are non-negative due to the rotation matrix symmetry: $∆\tilde{B}\left(\psi_{j},u_{j}\right)=∆\tilde{B}\left(-\psi_{j},-u_{j}\right)$. The median of these angles, $\hat{\psi }$, is reported as the metric.

For systems using N>3 markers, the orientation matrix $B_{j}$ can be determined by performing the Singular Value Decomposition (SVD) of the N×3 matrix, ${\left[b_{1,j}\ldots b_{N,j}\right]}^{T}$. Then, *N*-tuples of points $\left[b_{1,j},\ldots ,b_{N,j}\right]$ for j>1 are registered to the first *N*-tuple, and the remaining calculations are the same as for the N=3 case.

Note that the systematic bias in an uncalibrated sensor may affect the position component of the derived 6DOF pose differently than the orientation component. If the measured point, $b_{mes}=s\;b_{true}+t$ (where t is a constant vector and/or $s\left(\|b_{true}\|\right)\neq 1$ is a scalar function of distance ‖$b_{true}$‖), then the position component of the pose (i.e., centroid $b_{j}\;$) provided by the sensor is inaccurate, while the orientation matrix, $B_{j}$, in Equation (16) is still correct. However, when measurements are affected by the non-homogeneous scaling (i.e., the scale is no longer a scalar, but rather a tensor, and $b_{mes}=\left(s_{x}x_{true},\;s_{y}y_{true},\;s_{z}z_{true}\right)$), then both the orientation and position parts of the 6DOF pose are affected.

### 4.3. Metric 3: Difference of Angles of Relative Rotations

For any pair, $\left(A_{j},A_{k}\right)$, of orientations acquired in one coordinate frame (e.g., the GT’s coordinate system or, as in this case, the robot’s coordinate system), and the same pair of orientations, $\left(B_{j},B_{k}\right)$, measured in another coordinate system (for example, the sensor’s coordinate system), the relative rotations in axis u and angle ω representations are:

(20) $$R\left(\omega_{k,j}^{rob},u_{k,j}^{rob}\right)=\;A_{j}\;A_{k}^{T},$$

(21) $$R\left(\omega_{k,j}^{sen},u_{k,j}^{sen}\right)=\;B_{j}\;B_{k}^{T},$$

The difference between the angles:

(22) $${∆}_{k,j}\;=\;\;\omega_{k,j}^{sen}-\;\omega_{k,j}^{rob}.$$

accounts for the error in the relative rotations obtained in the two coordinate systems, {A} and {B}. Since this is a relative error, and not an absolute error, the calculation does not require registration between both coordinate systems. The difference ∆ can take both positive and negative values, and the standard deviation, $\sigma_{∆}$, is used as the third metric. Note that the angle of rotation, ω, is invariant to the coordinate frame (as the angular error ρ in Equation (10) and Equation (12)). Therefore, the difference ∆ in Equation (22) of the two angles characterizing rotations in two different coordinate systems is meaningful.

### 4.4. Metric 4: Variability of Orientations for Multiple Inverse Solutions of Robot Kinematics

Unlike the three general metrics described earlier, the fourth metric is specifically intended for evaluating pose measuring systems used in robotic applications. The angle error, ∆, in Equation (22) is defined as the difference between two corresponding angles of rotation in both the robot Equation (20) and sensor Equation (21) coordinate systems. Thus, both systematic biases originating from the sensor and robot contribute to the angle ∆, and, generally, it is hard to separate them and estimate their levels independently.

If both robot poses (j,k) in Equation (20) have their corresponding arm configurations $\left(\theta_{j},\theta_{k}\right)$ close to each other, then the angle of relative rotation, $\omega_{k,j}^{rob}$, in Equation (20) is only slightly affected by the robot calibration error. However, if the joint angles $\theta_{j}$ differ substantially from $\theta_{k}$, then $\omega_{k,j}^{rob}$ is more sensitive to the robot’s calibration error. To test this scenario, for each *j-*th robot pose in Cartesian space, all M(j) inverse kinematic solutions are calculated, and the robot is commanded to go to each of them. When the inaccurate parameters in the robot’s forward kinematic model are used, the resulting M(j) poses recorded by the external pose measuring system yield dispersion. If $B\left(\phi_{j,m},u_{j,m}\right)$ denotes the orientation part of the pose measured by the system (in angle-axis representation) for the *j-*th Cartesian pose and the *m-*th arm configuration, then the standard deviation $\sigma_{\phi }\left(j\right)$ of angles $\phi_{j,m}$ characterizes the spread in actual robot orientations. The median, ${\hat{\sigma }}_{\phi }$, is used as the fourth auxiliary metric to estimate the contributions of the pose measuring system and robot to the third metric, $\sigma_{∆}$.

## 5. Experimental Setup

Data for calculating the four metrics described in Section 4, and for evaluating the performance of the RRBC method using the errors defined in Equations (6) and (7), were acquired by two pose measuring systems along with an industrial robot arm in two different experiments described in Section 5.1 and Section 5.2.

An industrial, open-chain manipulator robot, the KUKA LWR 4+, was used in both experiments. Per the robot’s specification, the repeatability $\sigma_{rep}$ of this 7DOF robot arm is ± 0.05 mm (ISO 9283:1998). To ensure high accuracy in Cartesian space, the stiffness of the robot was set high. For all trials, the third joint angle of the robot was fixed and set to zero, reducing the robot to a 6DOF model. An in-house analytical inverse kinematic (AIK) module was used to solve the inverse kinematic problem rather than relying on the robot’s built-in IK solver. Depending on the pose of the end-effector, the AIK module yielded up to eight unambiguous solutions.

**Figure 1:**
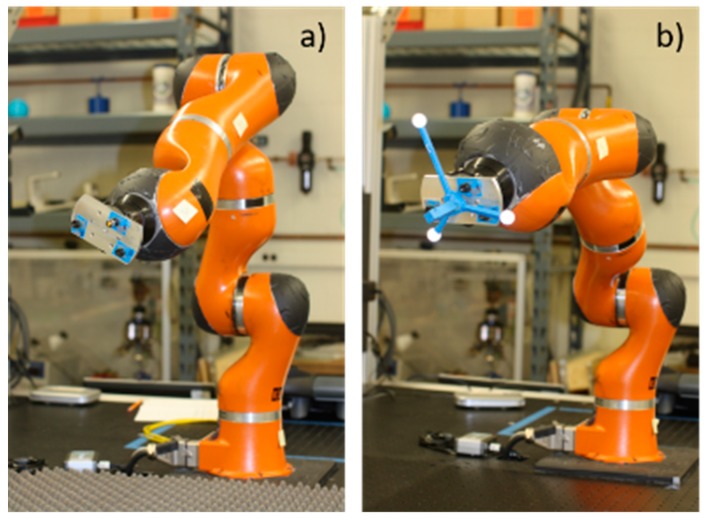
Robotic arm with two different sets of 3D markers used for pose determination: (**a**) a single SMR in one of three nests used for a laser tracker; and (**b**) a SIR marker configuration for the motion capture system.

The first pose measuring system (used in [16]) was a fixed, three camera motion capture system, an OptiTrack TRIO. Each of the three cameras of the TRIO has a resolution of 640 ×480 pixels, and the sampling frequency of the tracking system was set to 120 Hz. Spherical infrared reflector (SIR) markers were attached to an aluminum plate mounted at the robot’s tool flange, as shown in Figure 1b. For each robot pose, the system output $\left(x_{j},y_{j},z_{j}\right)$ coordinates of each marker from which the corresponding pose, $\left(B_{j},b_{j}\right)$, in the sensor coordinate system was calculated as in Equations (16)–(19). To filter out sensor noise, 12 repeated measurements were made with the TRIO system, and only the averaged locations of each SIR marker were used for pose determination. The second pose measuring system used in the experiment was an API T3 laser tracker. This sensor can measure pose using a 6DOF SmartTrack Sensor (STS) target, but this target was not used in this study. For compatibility with processing data from the TRIO sensor, three 12.7 mm (0.5 inch) nests were glued to a flange mounted on the end of the robot arm, as shown in Figure 1a. For each commanded robot pose, a SMR was placed in each of the nests, and 3D data were acquired with the laser tracker. Both the SMR nests and SIR markers were arranged such that the distances between any two markers were sufficiently different to enable unique identification of each marker. Each recorded point represented an average of 50 measurements, and the 6DOF pose was then calculated using Equation (16). Collecting data using the SMR is a labor-intensive process since, for each commanded robot pose, a SMR had to be manually moved from one nest to another. Therefore, compared to the TRIO, a smaller amount of data was acquired with the laser tracker.

### 5.1. Experiment 1

The robot was commanded to K different target poses. Around each target pose, $\left(A_{k},a_{k}\right)$, a few surrounding fiducial poses, $\left(A_{j},a_{j}\right)$, were defined. For each commanded pose, target or fiducial, corresponding measurements, $\left(B_{k},b_{k}\right)$ and $\left(B_{j},b_{j}\right)$, were acquired using the pose measuring system. Fiducial poses were used to calculate in Equations (4) and (5) the corrections for the exact orientation, $\Lambda_{j}$, and position, $\lambda_{j}$, which were then used to produce the estimated target corrections in Equations (6) and (7). For the TRIO, there were K=66 target poses scattered throughout the robot’s work volume, and each target pose was surrounded by 48 fiducial poses as described in [16]. For the laser tracker, K=50 target poses were selected in the robot’s work volume, and 16 fiducial poses surrounded each target. Fiducial poses were obtained by deviating the last four robot joint angles by small amounts [±2°, ±3°, ±3°, ±4°]. In total, 3,234 robot poses were acquired with the TRIO, and 850 poses were acquired using the laser tracker. Almost all fiducial arm configurations associated with a given target were close to the target configuration. However, for some fiducials, the AIK module could not provide an inverse solution close to the nearby target solution. For these, the robot’s joint configurations for those fiducials were different from the nearby target configurations.

### 5.2. Experiment 2

A different set of J poses scattered in the robot work volume was selected to provide data for Experiment 2. In each selected *j*-th pose, the AIK module was used to calculate all M(j) inverse kinematic solutions, and the robot was commanded to position the TCP at each. Different joint angles corresponding to the same Cartesian pose had substantially different configurations. When the inaccurate parameters in the robot’s forward kinematic model were used, the resulting M(j) robot orientations $B\left(\phi_{j,m},u_{j,m}\right)$, as assessed by the pose measuring system, yielded a dispersion. For each *j*-th pose (j=1,…,J), the standard deviation, $\sigma_{\phi }\left(j\right)$, of angles, $\phi_{j,m}$, was calculated as described in Section 4.4. To ensure reliable estimates of $\sigma_{\phi }\left(j\right)$, only poses with M(j) ≥4 solutions were selected for the calculations. All inverse solutions were determined for each of the J=1,370 Cartesian poses measured by the TRIO, and J=46 poses measured by laser tracker.

## 6. Results

**Figure 2:**
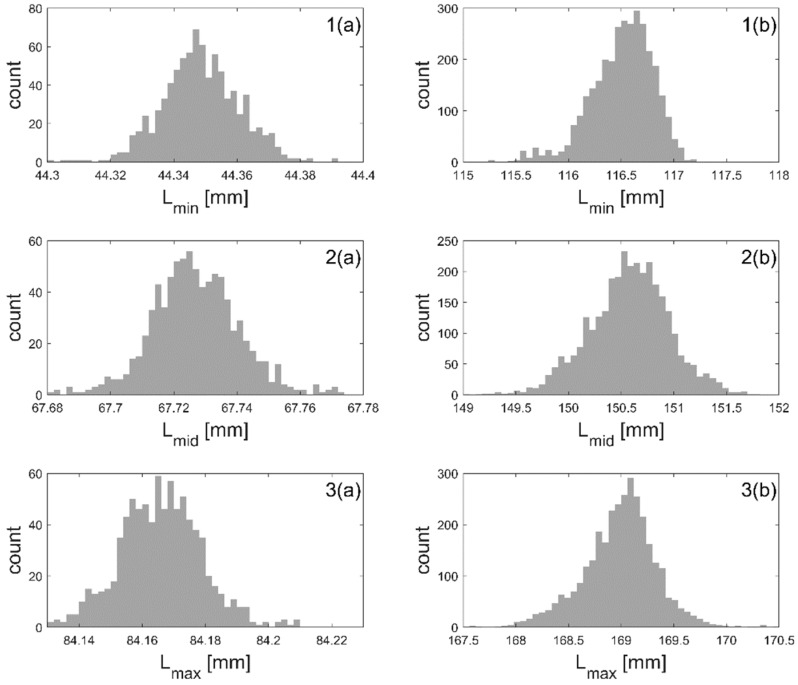
Histograms of the distances between the three markers measured in Experiment 1 by the laser tracker (left column) and the motion capture system (right column).

Histograms of the distances $\left(L_{min},\;\;L_{mid},\;\;L_{max}\right)$ between the three markers measured in Experiment 1 for the various robot arm poses are shown in Figure 2. The corresponding ratios of standard deviation to the mean $\sigma_{L}/\bar{L}$ (i.e., the first metric described in Section 4.1) are (0.03%, 0.04%, 0.02%) for the data acquired with the laser tracker, and (0.25%, 0.28%, 0.24%) with the motion capture system. Smaller ratios of all three distances indicate better compliance with the rigid-body assumption.

**Figure 3:**
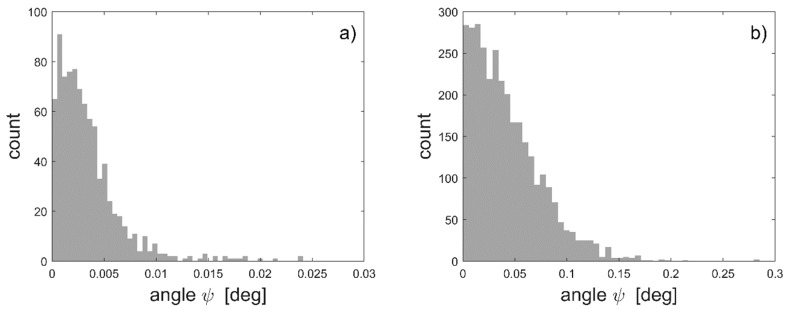
Histograms of angles ψ calculated from data obtained in Experiment 1 with: (**a**) laser tracker; (**b**) motion capture system.

Histograms of the angles ψ calculated from data acquired in Experiment 1 are shown in Figure 3. The median of these angles (i.e., the second metric described in Section 4.2) is $\hat{\psi }=0.003{}^{\circ}$ for data acquired with the laser tracker, and $\hat{\psi }=0.035{}^{\circ}$ with the motion capture system.

**Figure 4:**
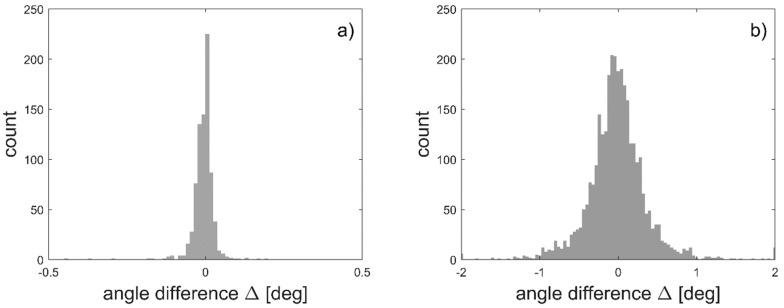
Histograms of angles ∆ calculated from data obtained in Experiment 1 with: (**a**) laser tracker; (**b**) motion capture system.

Histograms of angles Δ calculated from data acquired in Experiment 1 are shown in Figure 4. The standard deviation of these angles (i.e., the third metric described in Section 4.3) is $\sigma_{∆}=0.037{}^{\circ}$ for data acquired with the laser tracker, and $\sigma_{∆}=0.405{}^{\circ}$ for the motion capture system.

**Figure 5:**
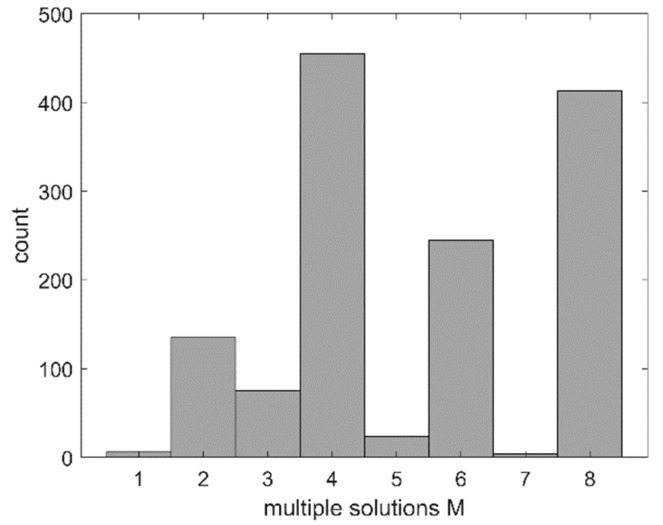
Histogram of the number of multiple solutions, M(j), provided by the AIK for each robot pose.

The histogram of the numbers M(j) of the inverse kinematic solutions for all J=1370 robot poses in Cartesian space obtained from data acquired in Experiment 2 is shown in Figure 5.

**Figure 6:**
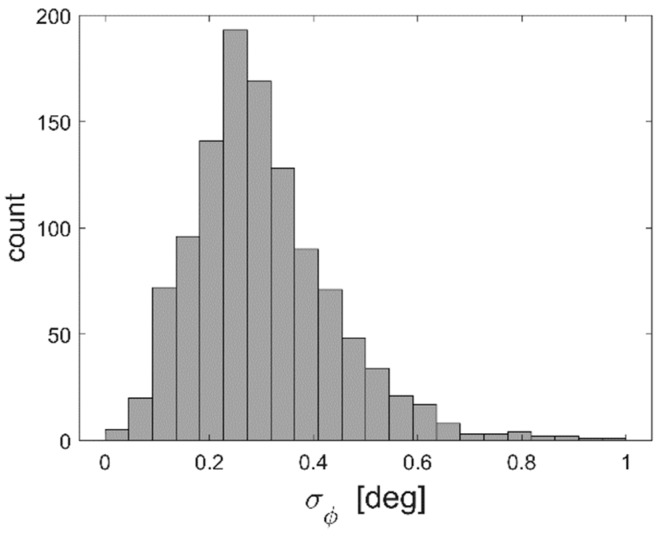
Histogram of standard deviations $\sigma_{\phi }$ gauging the spread of angles $\phi_{j,m}$ resulting from uncalibrated robot and multiple inverse kinematic solutions for data acquired in Experiment 2 with the motion capture system.

**Figure 7:**
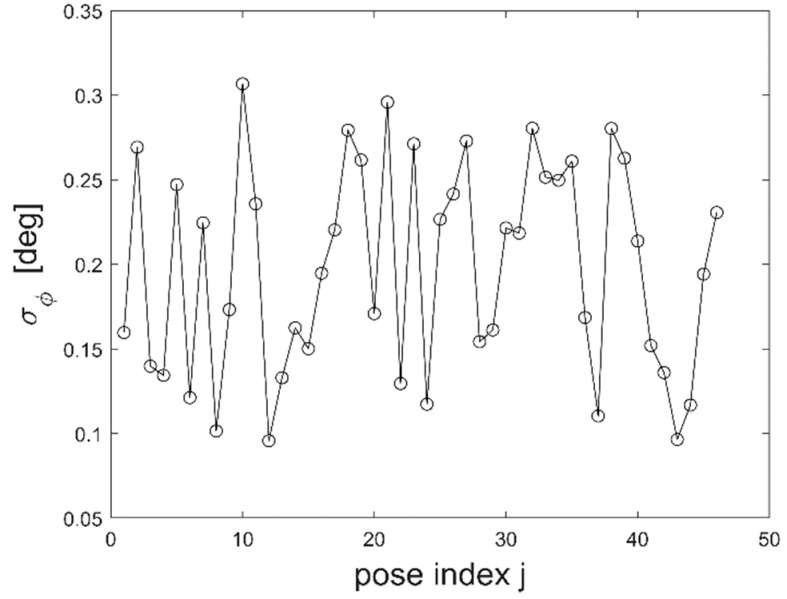
Standard deviations $\sigma_{\phi }$ gauging the spread of angles $\phi_{j,m}$ resulting from an uncalibrated robot and multiple inverse kinematic solutions from data acquired in Experiment 2 with laser tracker.

The histogram of standard deviations $\sigma_{\phi }\left(j\right)$ calculated from data acquired in Experiment 2 with the motion capture system is shown in Figure 6. The median of the J=1370 standard deviations (i.e., the fourth auxiliary metric described in Section 4.4) is ${\hat{\sigma }}_{\phi }=0.285{}^{\circ}$. For data acquired in Experiment 2 with the laser tracker, the median of the J=46 deviations, shown in Figure 7, is ${\hat{\sigma }}_{\phi }=0.168{}^{\circ}$.

**Figure 8:**
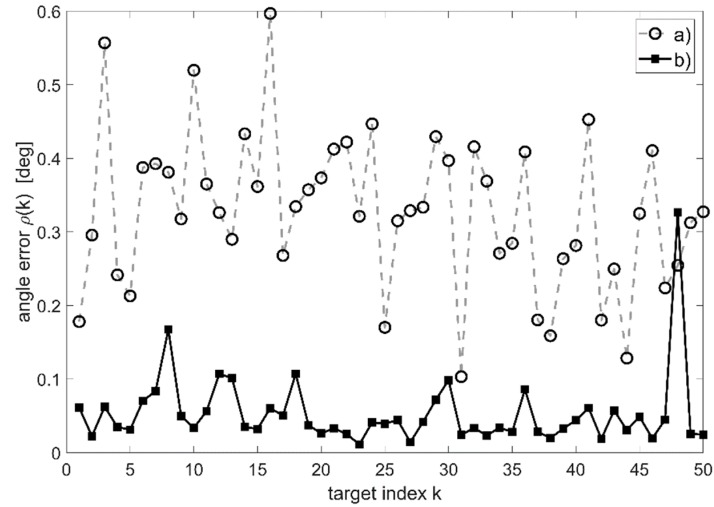
Target orientation error calculated from data acquired in Experiment 1 with the laser tracker: (**a**) uncorrected error $\rho_{unc}$; (**b**) corrected error $\rho_{cor}$.

**Figure 9:**
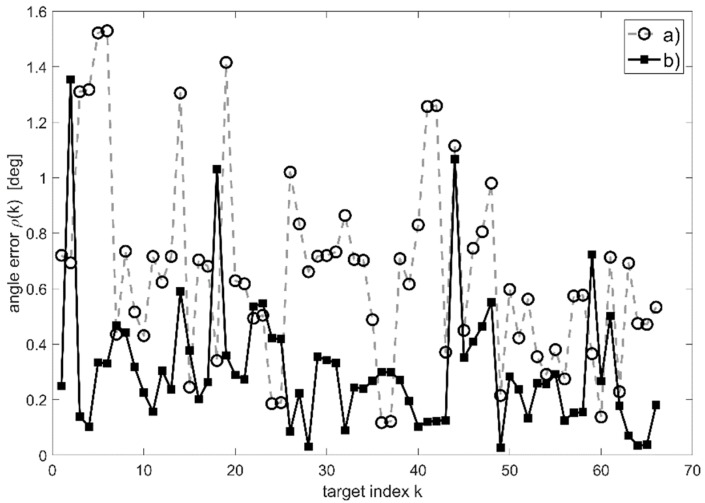
Target orientation error calculated from data acquired in [16] with the motion capture system: (**a**) uncorrected error $\rho_{unc}$; (**b**) corrected error $\rho_{cor}$.

The performance of the RRBC procedure was gauged by uncorrected and corrected orientation errors, $\left(\rho_{unc},\rho_{cor}\right)$, as defined in Equations (10) and (12), and the position errors, $\left(\delta_{unc},\delta_{cor}\right)$, as defined in Equations (11) and (13). Orientation errors obtained from the data acquired with the laser tracker in Experiment 1 are shown in Figure 8. For comparison, the orientation errors obtained with the motion capture system (presented earlier in [16]) are shown in Figure 9.

**Figure 10:**
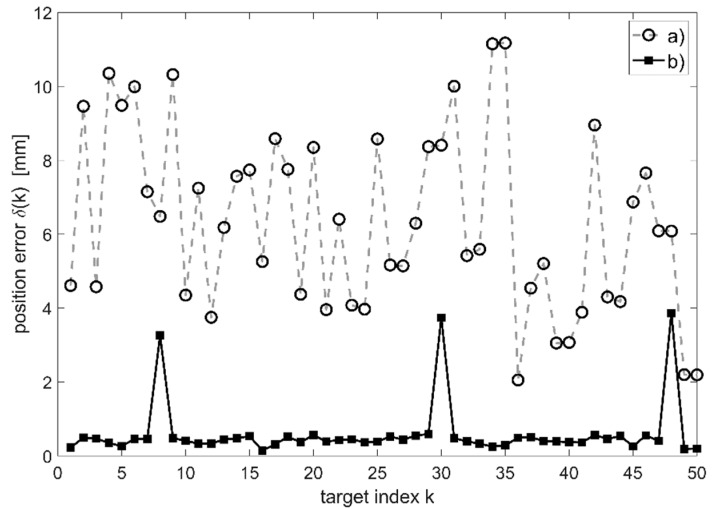
Target position error calculated from data acquired in Experiment 1 with the laser tracker: (**a**) uncorrected error $\delta_{unc}$; (**b**) corrected error $\delta_{cor}$.

**Figure 11:**
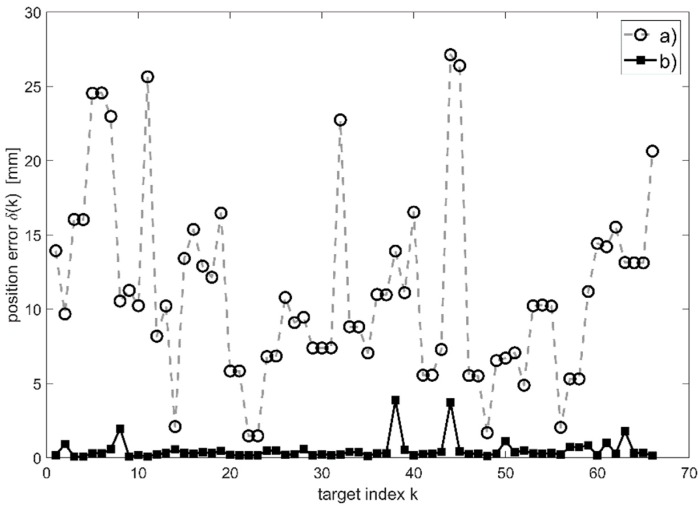
Target position error calculated from data acquired in [16] with the motion capture system: (**a**) uncorrected error $\delta_{unc}$; (**b**) corrected error $\delta_{cor}$.

The position errors obtained from the data acquired with the laser tracker in Experiment 1 are shown in Figure 10. For comparison, the position errors obtained with the motion capture system (presented earlier in [16]) are shown in Figure 11.

A summary of the results of the RRBC procedure applied to data acquired in Experiment 1 is provided in Table 1. The first two rows (A-exp and A-sim) are based on the motion capture system (experiment and simulation described in [16]), and the bottom row (B-exp) is based on data obtained with the laser tracker. The percentage reduction rates are calculated for the position and angular errors as;

(23) $$\gamma_{pos}=\;\left({\hat{\delta }}_{unc}-\;{\hat{\delta }}_{cor}\right)/{\hat{\delta }}_{unc},$$

(24) $$\gamma_{ang}=\;\left({\hat{\rho }}_{unc}-\;{\hat{\rho }}_{cor}\right)/{\hat{\rho }}_{unc},$$

where $\left({\hat{\delta }}_{unc},\;{\hat{\delta }}_{cor}\right)$ are the median target position errors shown in Figure 10 and Figure 11 for the uncorrected and RRBC corrected errors. Similarly, $\left({\hat{\rho }}_{unc},\;{\hat{\rho }}_{cor}\right)$ are the median target orientation errors shown in Figure 8 and Figure 9 for the uncorrected and RRBC-corrected errors.

## 7. Discussion

As seen from Figure 8, Figure 9, Figure 10 and Figure 11 and Table 1, two different results were obtained even though the same RRBC procedure was applied to reduce the localization errors of the same robot. The difference is therefore attributed to the use of different pose measuring systems.

The two systems belong to two different classes of instruments, which may be appropriate for different types of applications. The laser tracker yields measurements with lower uncertainty and higher accuracy than the motion capture system. Both systems derived 6DOF pose from the measurements of three 3D points. This approach requires that the configuration of the three points behaves as a rigid-body, which means that the distances between any two measured 3D points are fixed. While none of the systems used in the experiment fully complied with this requirement, the laser tracker provided data that better satisfied the rigid-body condition than the motion tracking system, as the plots in Figure 2, Figure 3 and Figure 4 imply. Consequently, we conclude that both the robot’s position and orientation errors can be reduced if more accurate 6DOF data is used to calculate position and orientation corrections in Equations (6) and (7). The three metrics defined in Section 4.1, Section 4.2 and Section 4.3, (i.e., the ratios $\sigma_{L}/\bar{L}$, the median angle $\hat{\psi }$, and the standard deviation $\sigma_{\Delta }$) were calculated from mean marker locations (average over 12 repeats for the motion capture system and 50 for the laser tracker). Therefore, the observed spread of ψ, ∆ and L is caused by systematic error (bias) that is dependent on robot position.

The metrics provide useful inputs when selecting a pose measuring system for robotic applications. To simultaneously reduce both position and orientation errors, the biases in the data acquired with the pose measuring system should be sufficiently small. The data shown in Figure 2 and Figure 3 indicate that, for systems deriving 6DOF pose from the measurements of a few points, the quality of the data used for reducing robot error should satisfy the condition $\sigma_{L}/\bar{L}\;<0.04\;\%$ for all distances L between any two markers, and the median angle $\hat{\psi }\;<0.003{}^{\circ}$.

A comparison of the angles ∆, shown in Figure 4, with the standard deviations $\sigma_{\phi }$, shown in Figure 6 and Figure 7, can help estimate the actual level of robot miscalibration. Both variables are affected by biases from the pose measuring systems and the uncalibrated robot. Recall that the angles ∆ were calculated from pairs of target-fiducial robot poses that had very similar arm configurations. In contrast, $\sigma_{\phi }$ was calculated using pairs of poses that had very different arm configurations. Because both ∆ and $\sigma_{\phi }$ are derived from relative rotations, it is reasonable to expect that inaccurate DH parameters would have a more significant effect on $\sigma_{\phi }$ than on ∆. However, this reasoning is blurred by differences in performance between the two pose measuring systems, gauged by $\sigma_{L}$ and $\hat{\psi }$. For the motion capture system, $\sigma_{L}$ and $\hat{\psi }$ are both large, and $\sigma_{∆}=0.405{}^{\circ}$ and ${\hat{\sigma }}_{\phi }=0.285{}^{\circ}$. For the laser tracker, $\sigma_{L}$ and $\hat{\psi }$ are both small, and $\sigma_{∆}=0.037{}^{\circ}$ and ${\hat{\sigma }}_{\phi }=0.204{}^{\circ}$. Therefore, we propose an estimate of the lower bound of the actual robot orientation error as $\chi =\;\left|\sigma_{∆}-\;{\hat{\sigma }}_{\phi }\right|$. For the motion capture system, χ=0.120° and for laser tracker χ=0.167°. In this case, since the tracker is a more accurate instrument than the motion capture system, the more reliable estimate of the robot orientation error is 0.167°.

While the laser tracker may provide more accurate measurements of 3D points than the camera-based motion capture system, it is not obvious that the more accurate positional measurements will always yield more accurate orientation data as characterized by smaller $\sigma_{∆}$ and $\hat{\psi }$. Both systems directly measure 3D points, and 6DOF pose is derived from these measurements. Thus, the quality of the orientation data depends not only on the bias in the acquired 3D points, but also on their relative placement in space (i.e., the distances L between each two points): larger distances yield better orientation data.

The two metrics ($\sigma_{L}/\bar{L}$ and $\hat{\psi }\;$) are based only on point measurements, and do not require a GT sensor or a specialized 6DOF target. Both provided useful information about marker-based pose measuring systems. In an ideal situation where the rigid-body condition is perfectly satisfied, $\sigma_{L}$ and $\hat{\psi }$ would be zero. Larger values of one or both parameters indicate worse performance. For example, when the measurement of a marker is affected by homogenous scaling, the derived orientation remains correct, and only the positional part of the 6DOF pose is affected. In this situation, the resulting $\hat{\psi }\approx 0{}^{\circ}$ while $\sigma_{L}/\bar{L}$ will be proportional to the scale factor. For non-homogenous scaling (i.e., when scale is a tensor) both orientation and position components of the 6DOF pose are affected. Then, both parameters $\hat{\psi }$ and $\sigma_{L}/\bar{L}$ will depend on the magnitude of scale.

Both angles, ψ and ∆ (i.e., metrics two and three), account for different aspects of the systematic error and a direct comparison of them can be misleading. Note that angles ψ shown in Figure 3 were calculated from triplets of 3D points acquired in different robot poses, and then transformed to the first frame using rigid-body registration. Since registration is based on a minimization of the error function, it is not surprising that the spread of ψ in Figure 3 is smaller than the spread of angles ∆ (which do not require any minimization) shown in Figure 4. Thus, angles ψ should be interpreted as a lower bound of the differences ∆ and both could be used in a complementary way to evaluate the relative performance of two or more pose measuring systems.

In our previous study [16] (based only on data acquired with a motion capture system), we reported that only the position median error was substantially reduced (97%), while the orientation median error was reduced only by 57%. Even more troubling was the observation that, for some robot poses, the corrected orientation error (after application of the RRBC method) was actually larger than the uncorrected error. For the dataset acquired with the laser tracker in the current study, all but one target pose (#48 in Figure 8) resulted in a corrected orientation error smaller than the uncorrected one. It is noted that the reduction rate, $\gamma_{ang}$, reported for this dataset (B-exp in Table 1) is closer to the simulation results presented in [16] (A-sim in Table 1). This is expected as the simulated and laser tracker data better satisfied the rigid-body condition than the motion capture data.

The results given in Table 1 indicate that the quality of the collected data more significantly affects the reduction of the orientation error, ρ, than the reduction of the position error, δ. This is not surprising because the position component of the 6DOF pose provided by the sensor is defined as the centroid of three 3D points. Any deviations from the rigid-body condition is mitigated by averaging the locations of the three measured points. No such mitigating mechanism exists for the orientation component of the full pose, which makes reduction of the orientation error more vulnerable to the biases in the measurement of the 3D points.

As mentioned earlier, most of the fiducial poses surrounding each *k*-th target had robot arm configurations close to the configuration of the target pose. However, for some targets, this condition could not be satisfied, and the joint angles of the fiducial configurations were substantially different from the target joint angles, even though the corresponding poses in the Cartesian space were close. In such rare configurations, the robot calibration error had more of an impact, and led to a degraded performance of linear interpolation of the estimated target corrections in Equations (6) and (7). Consequently, large outliers are seen in the corrected position error, $\delta_{cor}\left(k\right)$, and orientation error, $\rho_{cor}\left(k\right)$ (see, for example, k=48 in Figure 8 and Figure 10).

In summary, the metrics proposed in this paper helped to explain the conflicting outcomes of the reduction of robot localization error. Two of these metrics are convenient as they gauge the performance of marker-based systems by evaluating a deviation from the rigid-body condition. Thus, they do not require ground truth sensors. It remains an open question as to how to develop similar metrics for other, markerless pose measuring systems.

## Figures and Tables

**Table 1:** Median robot errors.

Data Source	Median Position Error [mm]			Median Angle Error [deg]		
	$\;{\hat{\delta }}_{unc}\;$	$\;{\hat{\delta }}_{cor}\;$	$\;\gamma_{pos}\;$	$\;{\hat{\rho }}_{unc}\;$	$\;{\hat{\rho }}_{cor}\;$	$\;\gamma_{ang}\;$
A-exp	10.233	0.301	97%	0.626	0.267	57%
A-sim	9.128	0.228	97%	0.952	0.010	99%
B-exp	6.134	0.433	92%	0.327	0.038	88%
